# Benzodiazepine use in relation to long-term dementia risk and imaging markers of neurodegeneration: a population-based study

**DOI:** 10.1186/s12916-024-03437-5

**Published:** 2024-07-02

**Authors:** Ilse vom Hofe, Bruno H. Stricker, Meike W. Vernooij, M. Kamran Ikram, M. Arfan Ikram, Frank J. Wolters

**Affiliations:** 1 Department of Epidemiology, Erasmus University Medical Center, Rotterdam, The Netherlands; 2 Department of Radiology & Nuclear Medicine, Alzheimer Centre Erasmus MC, Erasmus University Medical Center, Rotterdam, The Netherlands; 3 Department of Neurology, Erasmus University Medical Center, Rotterdam, The Netherlands

**Keywords:** Benzodiazepine use, Dementia, MRI, Population-based

## Abstract

**Background:**

Benzodiazepine use is common, particularly in older adults. Benzodiazepines have well-established acute adverse effects on cognition, but long-term effects on neurodegeneration and dementia risk remain uncertain.

**Methods:**

We included 5443 cognitively healthy (MMSE ≥ 26) participants from the population-based Rotterdam Study (57.4% women, mean age 70.6 years). Benzodiazepine use from 1991 until baseline (2005–2008) was derived from pharmacy dispensing records, from which we determined drug type and cumulative dose. Benzodiazepine use was defined as prescription of anxiolytics (ATC-code: N05BA) or sedative-hypnotics (ATC-code: N05CD) between inception of pharmacy records and study baseline. Cumulative dose was calculated as the sum of the defined daily doses for all prescriptions. We determined the association with dementia risk until 2020 using Cox regression. Among 4836 participants with repeated brain MRI, we further determined the association of benzodiazepine use with changes in neuroimaging markers using linear mixed models.

**Results:**

Of all 5443 participants, 2697 (49.5%) had used benzodiazepines at any time in the 15 years preceding baseline, of whom 1263 (46.8%) used anxiolytics, 530 (19.7%) sedative-hypnotics, and 904 (33.5%) used both; 345 (12.8%) participants were still using at baseline assessment. During a mean follow-up of 11.2 years, 726 participants (13.3%) developed dementia. Overall, use of benzodiazepines was not associated with dementia risk compared to never use (HR [95% CI]: 1.06 [0.90–1.25]), irrespective of cumulative dose. Risk estimates were somewhat higher for any use of anxiolytics than for sedative-hypnotics (HR 1.17 [0.96–1.41] vs 0.92 [0.70–1.21]), with strongest associations for high cumulative dose of anxiolytics (HR [95% CI] 1.33 [1.04–1.71]). In imaging analyses, current use of benzodiazepine was associated cross-sectionally with lower brain volumes of the hippocampus, amygdala, and thalamus and longitudinally with accelerated volume loss of the hippocampus and to a lesser extent amygdala. However, imaging findings did not differ by type of benzodiazepines or cumulative dose.

**Conclusions:**

In this population-based sample of cognitively healthy adults, overall use of benzodiazepines was not associated with increased dementia risk, but potential class-dependent adverse effects and associations with subclinical markers of neurodegeneration may warrant further investigation.

**Supplementary Information:**

The online version contains supplementary material available at 10.1186/s12916-024-03437-5.

## Background

Benzodiazepines are the most commonly prescribed psychotropic medication in developed countries, and the number of prescriptions is increasing [1, 2]. Approximately 10% of the adult European population is using benzodiazepines, increasing with age up to 30% in people aged 65 years or older [1, 3]. Guidelines discourage long-term use due to risk of psychological and physical dependence, falls, and cognitive impairment, especially in older adults. Nevertheless, approximately 30–40% of older benzodiazepine users continues use beyond the recommended period of several weeks [4, 5]. This increasing trend of prolonged use of benzodiazepines raises concerns about potential long-term adverse effects, in particular on cognitive ability and dementia risk.

Benzodiazepine use has well-established acute effects on cognition through GABAergic effects, which may persist long after withdrawal [6]. Long-term effects, however, are uncertain and need not be purely detrimental. On the basis of animal studies, benzodiazepine exposure might have neuroprotective effects through reduced neuroinflammation and mitigating ApoE-induced phosphorylation and dysregulation of hippocampal neurogenesis as well as detrimental effects on dementia pathology through augmentation of tau phosphorylation, amyloid deposition, and reduction of brain-derived neurotropic factor (BDNF) [7]. Several observational studies have investigated whether these observations translate also into altered dementia risk in humans. Results from two recent meta-analyses suggest that use of benzodiazepines is associated with higher dementia risk, indicating that the harmful effects of benzodiazepine might outweigh any protective effects. However, causal inference from the included studies was hampered by methodological and statistical heterogeneity [8, 9]. Reversed causation, confounding by indication, and residual confounding in particular raised doubt about the causal interpretation of observations. Anxiety and sleep disturbances are suggested to be independent risk factors for dementia [10, 11], and consequent prescription of benzodiazepine medication against anxiety or sleep disturbances may lead to spurious associations between benzodiazepine use and dementia (i.e., confounding-by-indication). Moreover, symptoms of anxiety and sleep disturbances frequently occur in the prodromal phase of dementia, in response to perceived decline in cognitive ability or as a consequence of shared neurobiological pathways between dementia, anxiety, and sleep disorders. In this context too, spurious associations can arise due to prescription of benzodiazepines in response to prodromal features of dementia, a phenomenon commonly referred to as reversed causation [12]. Heterogeneity may be increased due to differential effects of benzodiazepines on Alzheimer’s disease pathology versus other causes of neurodegeneration [13]. Concurrent assessment of preclinical markers of dementia, notably imaging markers, could in part alleviate these concerns and provide further insight in the neurobiological mechanisms through which benzodiazepine use might increase dementia risk, but such studies are scarce. One cross-sectional study among 2323 patients attending a French memory clinic found that benzodiazepine users had *larger* hippocampal volumes [14], but no published studies have determined the long-term association between benzodiazepine use and preclinical neurodegeneration in unselected populations.

We hypothesized that long-term use of benzodiazepines affects measures of structural brain imaging as well as long-term dementia risk. We therefore aimed to determine the effect of benzodiazepine use on long-term dementia risk and on imaging markers of neurodegeneration in a prospective, population-based cohort of cognitively healthy older adults.

## Methods

### Study population

Data was drawn from the Rotterdam Study, of which details have been described elsewhere [15]. In short, the Rotterdam Study is an ongoing prospective population-based cohort study, which started in 1990 with the aim to investigate the occurrence and determinants of common diseases in the elderly [15]. In 1990, the Rotterdam Study started with an original cohort of 7983 participants aged 55 years and older (RS-I). In 2000, this cohort was extended with 3011 participants who had reached age 55 or moved into the study area (RS-II). In 2006, an additional 3932 participants aged 45 years and over were included (RS-III), which resulted in a total study population of 14,926 participants. Participants undergo follow-up examinations every 4 years at a dedicated research center. For the incident dementia analyses in the current study, we included participants aged 60 years or older who took part in the fourth visit of RS-I (2002–2004), the second visit of RS-II (2004–2005), or the first visit of RS-III (2006–2008). Of 6258 eligible participants, we excluded those with cognitive impairment (Mini-Mental State Examination (MMSE) < 26) at baseline (*N* = 806), those with missing pharmacy data (*N* = 8), and those who withdrew informed consent for dementia follow-up (*N* = 1), which resulted in the inclusion of 5443 participants. An overview of the inclusion of participants is presented in Additional file 1: Figure S1. Brain magnetic resonance imaging (MRI) was incorporated in the Rotterdam Study protocol from 2005 onwards. For the MRI analyses, we included cognitively healthy (MMSE ≥ 26) participants who underwent brain MRI between 2005 and 2015. Given the long preclinical phase of neurodegenerative disease, all persons > 45 years were allowed to participate. Of 4956 eligible participants who underwent brain MRI, 4836 had at least one scan that passed quality control.

### Use of benzodiazepines and Z-drugs

Information on benzodiazepine use was available through pharmacy dispensing records from 1991 onwards for cohort RS-I, and from 1995 onwards for cohort RS-II and cohort RS-III, classified according to the Anatomical Therapeutic Chemical (ATC) code. We extracted all filled prescriptions of benzodiazepines from inception of pharmacy records to study baseline (2002–2008). For each prescription, we extracted the prescription date, duration of use, and strength in defined daily dosage (DDD), as defined by the World Health Organization [16]. Benzodiazepine use was defined as prescription of anxiolytics (ATC-code: N05BA) or sedative-hypnotics (ATC-code: N05CD) between inception of pharmacy records and study baseline. We further calculated cumulative defined daily dose from inception of pharmacy records to baseline and whether participants were still using at baseline or discontinued use prior to baseline. Similarly, information on the use of Z-drugs (ATC-code: N05CF) was extracted.

### Dementia screening and surveillance

Participants were screened for dementia at each center visit, using the Mini-Mental State Examination (MMSE) and the Geriatric Mental Schedule (GMS). Those with MMSE < 26 or GMS > 0 underwent further investigation, including an informant interview and the Cambridge Examination for Mental Disorders of the Elderly (CAMDEX). In addition, the entire cohort was continuously under surveillance for dementia through electronic linkage with medical records from general practitioners and the regional institute for outpatient mental health care. All cases suspect for dementia were reviewed by a consensus panel, led by a consultant neurologist, which applied standard criteria for dementia (Diagnostic and Statistical Manual of Mental Disorders (DSM)-III-R) to come to a final diagnosis. Participants were censored at date of dementia diagnosis, date of death, date of loss to follow-up, or January 1, 2020, whichever came first [17]. Follow-up for dementia until 1 January 2020 was complete for 93.9% of the potential person years.

### MRI protocol and image processing

MRI of the brain was performed on a 1.5 T scanner (General Electric Healthcare, Milwaukee, WI) using an 8-channel head coil. Imaging acquisition included a high-resolution 3D T1-weighted, proton density-weighted, and a fluid-attenuated inversion recovery (FLAIR) sequence. A detailed scan protocol of the Rotterdam Study is described elsewhere [18]. Volumes in milliliters (mL) of the total brain, grey matter, and white matter were obtained by automated tissue segmentation based on a k-nearest neighbor algorithm. All segmentations were visually inspected and manually corrected when necessary. Volumes of subcortical structures involved in memory and mood regulation (i.e., the hippocampus, thalamus and amygdala) were obtained by processing T1-weighted images with FreeSurfer (version 6.0) [19].

### Other measurements

Information on age, sex, educational attainment (primary, lower, intermediate or higher education), smoking habits (never, current, or former), and alcohol use (grams/day) was ascertained during a home interview. Prevalence of stroke, cancer, coronary heart disease, congestive heart failure, atrial fibrillation, and chronic obstructive pulmonary disease was assessed by interview at baseline and verified in medical records. The Composite International Diagnostic Interview (CIDI) [20] was used for the assessment of symptoms of anxiety; the Pittsburg Sleep Quality Index (PSQI) [21] was used to assess sleep quality. Presence of depressive symptoms was defined as a score of > 15 on the Center for Epidemiology Depression Scale (CES-D) [22] or the use of antidepressants. During baseline center visit, blood pressure was measured in sitting position using a random-zero sphygmomanometer; hypertension was defined as a systolic blood pressure > 140 mmHg, a diastolic blood pressure > 90 mmHg, or the use of blood pressure-lowering medication. The estimated glomerular filtration rate (eGFR) was calculated using the Chronic Kidney Disease Epidemiology Collaboration equation, based on creatinine concentrations in fasting blood samples [23]. Diabetes was defined as fasting blood glucose > 7.0 mmol/L or use of antidiabetic medication. Total fat mass was obtained using dual-energy X-ray absorptiometry (DXA) scans.

### Statistical analyses

Missing covariate data were imputed using tenfold imputation. Distribution of variables was similar in the imputed and non-imputed datasets. Percentages of missing data are shown in the footnote of Table 1. Data on all variables were at least 90% complete, except for fat mass (73%). For the main analyses, ever use of benzodiazepines was compared to never use. In secondary analyses, we distinguished former from current use, stratified by the median cumulative defined daily dose, and differentiated anxiolytic from sedative-hypnotic benzodiazepines.

First, we determined the association between benzodiazepine use and risk of all-cause dementia using Cox proportional hazards regression models. All analyses were adjusted for age, sex, education, and time between inception of pharmacy records and baseline visit (model 1) and additionally for smoking habits, alcohol use, total fat mass, eGFR, presence of symptoms of anxiety, sleep problems or depression, and prevalence of diabetes, stroke, atrial fibrillation, congestive heart failure, coronary heart disease, cancer, and chronic obstructive pulmonary disease (model 2). In various sensitivity analyses, we then (i) stratified by presence of at least one anxiety disorder according to a score above the cut-off on the CIDI; (ii) stratified by the presence of sleep problems according to the PSQI; (iii) stratified on high vs. low alcohol use, where high alcohol use was defined as average consumption of more than 2 units (10 g) per day; and (iv) included benzodiazepine use as a time-varying variable up till dementia diagnosis. We compared the associations of oxazepam (*t*_1/2_ = 5–15 h) and diazepam (*t*_1/2_ = 20–70 h) with dementia risk to assess the effect of drug half-life. Finally, we assessed the effect of use of Z-drugs on dementia risk.

Next, we determined baseline differences in brain volumes between benzodiazepine users and non-users using linear regression models and applied linear mixed models to determine the association between benzodiazepine use and change in brain volumes over time of the total brain, white matter, grey matter, hippocampus, amygdala, and thalamus. Adjustments were similar to the dementia models, with the addition of total intracranial volume. To account for possible nonlinear trajectories, we included splines of follow-up time, with knots at the median follow-up duration of 3.3 years. An interaction of follow-up time with age was included, to allow for slope differences in the relationship with age.

Analyses were done using SPSS version 28 [24] and R version 4.1.3 (packages: “Mice,” “nlme”).

### Patient and public involvement

Participants of the Rotterdam Study are represented through a panel that is consulted on a regular basis about study management and results. All participants are informed on results and publications of the Rotterdam Study through newsletters. In the current manuscript, participants were not involved in the development of research questions or study design.

## Results

Table 1 contains baseline characteristics of the study population. During the exposure period preceding baseline, 2701 (49.6%) participants had used benzodiazepines at any time, of whom 1264 (46.7%) had exclusively used anxiolytics, 533 (19.7%) had used sedative-hypnotics, and 904 (33.5%) had used both. In total, 368 (7.8%) had used Z-drugs, of whom 306 (83.2%) also used benzodiazepines. At study baseline, 345 (12.8%) participants were presently using benzodiazepines.

**Table 1:** Baseline characteristics of the study population

**Characteristics**	**Total study population** (*N* = 5443)	**MRI sample** (*N* = 4836)
Age, years	70.6 (± 7.6)	63.4 (± 11.1)
Women	3125 (57.4%)	2694 (55.7%)
Education		
Primary	531 (9.8%)	378 (7.8%)
Lower or intermediate vocational	2343 (43.0%)	1806 (37.3%)
Intermediate vocational or higher	1639 (30.1%)	1476 (30.5%)
Higher vocational or university	859 (15.8%)	1129 (23.3%)
Anxiety^a^	391 (7.2%)	324 (6.7%)
Sleep quality^b^	3.0 (1.2–6.0)	3.0 (2.0–6.0)
Depressive symptoms^c^	1163 (21.4%)	551 (11.4%)
Fat mass, kg	26.9 (± 9.0)	26.9 (± 8.9)
Smoking		
Never	1559 (28.6%)	1444 (29.9%)
Former	2760 (50.7%)	2326 (48.1%)
Current	899 (16.5%)	975 (20.2%)
Alcohol, grams/day	7.4 (0.71–20.0)	1.6 (1.0–8.57)
Diabetes	701 (12.9%)	596 (12.3%)
Hypertension	4161 (76.4%)	2997 (62.0%)
Stroke	209 (3.8%)	180 (3.7%)
Coronary heart disease	499 (9.2%)	300 (6.2%)
Heart failure	229 (4.2%)	59 (1.2%)
Atrial fibrillation	309 (5.7%)	136 (2.8%)
Cancer	513 (9.4%)	482 (10.0%)
Chronic obstructive pulmonary disease	422 (7.8%)	273 (5.6%)
Glomerular filtration rate	74.8 (± 14.5)	81.7 (± 15.6)

Data are presented as frequency (%) for categorical variables, mean ± standard deviation for normally distributed continuous variables, and median ± quartiles for non-normally distributed continuous variables

Covariates with missing data in total study population: education (1.3%), anxiety disorder (4.9%), poor sleep quality (3.4%), depression (3.5%), fat mass (27.7%), smoking (4.1%), alcohol (1.8%), diabetes (3.1%), hypertension (0.2%), coronary heart disease (1.6%), heart failure (0.1%), atrial fibrillation (8.1%), cancer (9.4%), chronic obstructive pulmonary disease (7.8%), glomerular filtration rate (5.2%)

^a^Presence of anxiety according to the Composite International Diagnostic Interview

^b^Sleep quality as assessed by the Pittsburg Sleep Quality Index

^c^Depression was defined as a score of > 15 on the Center for Epidemiology Depression Scale (CES-D) or the use of antidepressants

In participants aged < 55 years, 2.6% was currently using benzodiazepines at baseline, which increased up to 7.5% in those aged > 80 years. Regarding overall use during the exposure period, women more often had used benzodiazepines than men (57.2% vs. 39.4%), and use was also more frequent in lower educated individuals (55.2% in primary education vs. 41.6% in higher vocational education or university).

Cumulative dose of benzodiazepines until baseline was a median 37.0 defined daily dose [interquartile range (IQR): 10.0–240.0], which was lower for users of anxiolytics than for sedative-hypnotics (12.0 [5.0–53.0] vs. 35.0 [10.0–192.0], *p* < 0.001).

### Incident dementia

**Fig. 1:**
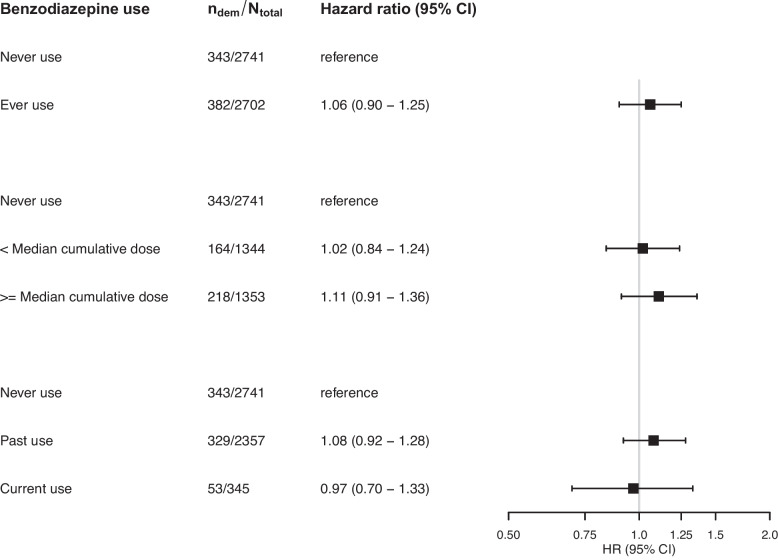
Benzodiazepine use and dementia risk. All estimates refer to the comparison with never use. Estimates are adjusted for age, sex, education, time between inception of pharmacy records and baseline visit, presence of anxiety, sleep problems and depression, smoking status, alcohol use, glomerular filtration rate, and prevalence of diabetes, hypertension, stroke, coronary heart disease, heart failure, atrial fibrillation, cancer, and chronic obstructive pulmonary disease. *N*_dem_ = number of dementia cases. *N*_total_ = total number of participants in group. CI, confidence interval. Median cumulative daily dose in any type was 37 DDD, in anxiolytics 18 DDD, and in sedative-hypnotics 51.5 DDD

During a mean follow-up of 11.2 years, 726 (13.3%) participants developed dementia. In fully adjusted models, use of benzodiazepines was not associated with dementia risk (hazard ratio [95% confidence interval]: 1.06 [0.90; 1.25]), irrespective of cumulative dose (Fig. 1). Effect estimates were similar between past users and those who were still using at baseline (Fig. 1).

**Fig. 2:**
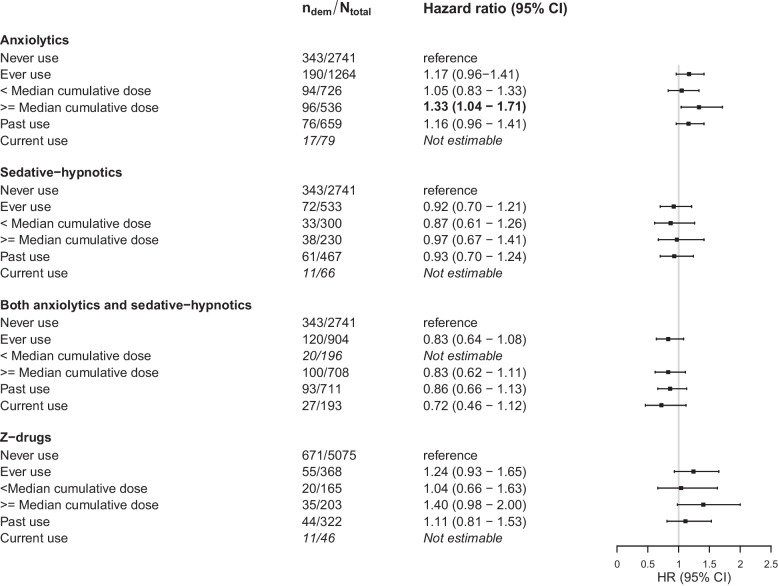
Types of benzodiazepines and Z-drugs and dementia risk. All estimates refer to the comparison with never use. Estimates are adjusted for age, sex, education, time between inception of pharmacy records and baseline visit, presence of anxiety, sleep problems and depression, smoking status, alcohol use, glomerular filtration rate, and prevalence of diabetes, hypertension, stroke, coronary heart disease, heart failure, atrial fibrillation, cancer, and chronic obstructive pulmonary disease. *N*_dem_ = number of dementia cases. *N*_total_ = total number of participants in group. CI, confidence interval. Median cumulative daily dose in any type was 37 DDD, in anxiolytics 18 DDD, in sedative-hypnotics 51.5 DDD, and in Z-drugs 30.0 DDD

Regarding different types of benzodiazepines, effect estimates were somewhat higher for ever use of anxiolytics than for sedative-hypnotics, although neither was statistically significant (Fig. 2). The highest risk estimates were observed for high cumulative dose of anxiolytics only (HR [95% CI] with low cumulative dose: 1.05 [0.83; 1.33] vs. high cumulative dose: 1.33 [1.04; 1.71]). No dose–response association was observed for sedative-hypnotics or for individuals with combined use of anxiolytics and sedative-hypnotics (Fig. 2). In sensitivity analyses assessing the two most commonly used types of anxiolytics, we observed no differences in effect estimates between the use of short half-life oxazepam and long half-life diazepam (ever use compared to never use, for oxazepam: HR [95% CI] 1.01 [0.78; 1.31] and for diazepam: HR [95% CI] 1.06 [0.82; 1.39]).

Presence of anxiety, poor self-reported sleep, and depressive symptoms were all more common in current users of benzodiazepines, compared to past users and in particular never users (Additional file 2: Table 1). Among 391 participants with high levels of anxiety at baseline, risk estimates for anytime benzodiazepine use were somewhat higher compared to those with low anxiety (with high anxiety; (HR [95% CI] 1.47 [0.78; 2.79]; with low anxiety: HR [95% CI] 1.05 [0.88; 1.25]). We observed opposite and less pronounced differences comparing those with and without sleep problems (Additional file 2: Table S1). Stratification on low alcohol consumption (average daily alcohol consumption below 2 units) vs. high alcohol consumption (average daily alcohol consumption of 2 units or higher) showed no difference in effect estimates in high alcohol consumption compared to low alcohol consumption (low alcohol consumption: 1.06 [0.88; 1.28], high alcohol consumption: 1.08 [0.73; 1.59], Additional file 2: Table S1), with a non-significant interaction between benzodiazepine and alcohol use (*p* = 0.411). Analyses including benzodiazepine use as time-varying exposure up till dementia diagnosis showed higher estimates in ever users (HR [95% CI] 1.22 [1.04; 1.43]).

For Z-drugs, ever use was not significantly associated with dementia risk (HR [95% CI] 1.24 [0.93; 1.65]). Effect estimates were somewhat higher for high cumulative dose (Fig. 2).

### Change in neuroimaging markers

Among 4836 participants with brain MRI, any use of benzodiazepines during the exposure period was not associated with brain volumes at baseline (Additional file 3: Table S2), but current use at baseline was significantly associated with lower total brain volume as well as volumes of the hippocampus, amygdala, and thalamus (Additional file 3: Table S2).

**Fig. 3:**
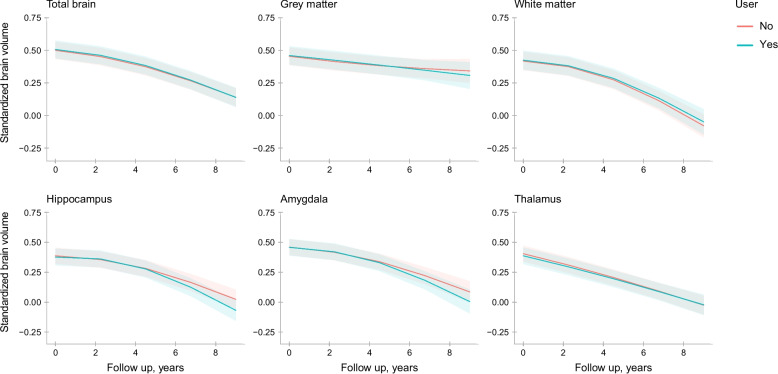
Benzodiazepine use and trajectories of standardized brain volumes during follow-up. For graphical representation, trajectories are depicted for mean age, sex, education, and time between inception of pharmacy records and scan date. Corresponding effect estimates in the main text refer to the fully adjusted models

Of all 4836 participants who underwent brain MRI, 3099 (64.1%) had at least 1 follow-up scan. Use of benzodiazepines was associated with accelerated reduction in hippocampal volume during follow-up, with most pronounced differences during long-term follow-up (change in standardized brain volume (*β*) [95% CI] in < 0–3 years: − 0.018 [− 0.060; 0.024] vs. > 3–10 years of follow-up: − 0.117 [− 0.211; − 0.023]; Fig. 3). A similar trend was observed in the amygdala (*β* [95% CI] in < 0–3 years of follow-up: − 0.035 [− 0.094; 0.024] vs. > 3–10 years of follow-up: − 0.101 [− 0.235; − 0.032]; Fig. 3). However, we observed no dose–response relationship in these associations, and in contrast to the cross-sectional imaging analyses, risk estimates were higher with former use than current use at baseline (Additional file 4: Table S3). No significant associations were observed with change in total brain volume nor with change in volumes of the grey matter, white matter, and thalamus (Fig. 3).

When assessing types of benzodiazepines separately, effect estimates for change in hippocampal and amygdalar volumes were comparable between anxiolytic and sedative-hypnotic use (Table 2). Across subtypes, once again, there were no significant associations of benzodiazepine use with change in total brain volume, grey matter volume, or thalamic volume. Use of anxiolytics was associated with *less* reduction in white matter volume, whereas combined use of anxiolytics and sedative-hypnotics was associated with *accelerated* reduction in white matter (Table 2). Use of Z-drugs was not associated with change in brain volume in any of the aforementioned brain areas (Table 2).

**Table 2:** Types of benzodiazepine and change in standardized brain volumes per year during follow-up

	**Brain region**	**1st spline: 0–3 years of follow-up**	**2nd spline: 3–10 years of follow-up**
		Mean difference (95% CI)	Mean difference (95% CI)
Anxiolytics	Total brain	0.010 (− 0.020; 0.040)	− 0.009 (− 0.076; 0.057)
	Grey matter	− 0.051 (− 0.129; 0.026)	− 0.118 (− 0.295; 0.060)
	White matter	0.074 (0.006; 0.142)	0.118 (− 0.035; 0.270)
	Hippocampus	− 0.016 (− 0.071; 0.039)	− 0.106 (− 0.228; 0.015)
	Amygdala	− 0.046 (− 0.122; 0.030)	− 0.081 (− 0.253; 0.090)
	Thalamus	0.006 (− 0.047; 0.059)	0.000 (− 0.117; 0.118)
Sedative-hypnotics	Total brain	0.007 (− 0.039; 0.054)	0.003 (− 0.093; 0.099)
	Grey matter	0.010 (− 0.114; 0.134)	0.058 (− 0.204; 0.319)
	White matter	0.005 (− 0.105; 0.114)	− 0.043 (− 0.271; 0.184)
	Hippocampus	− 0.003 (− 0.090; 0.083)	− 0.109 (− 0.286; 0.069)
	Amygdala	0.022 (− 0.098; 0.142)	− 0.088 (− 0.341; 0.164)
	Thalamus	0.034 (− 0.049; 0.117)	0.065 (− 0.107; 0.236)
Anxiolytics and sedative-hypnotics	Total brain	− 0.017 (− 0.053; 0.020)	− 0.026 (− 0.101; 0.049)
	Grey matter	0.062 (− 0.036; 0.160)	0.011 (− 0.195; 0.218)
	White matter	− 0.089 (− 0.176; − 0.003)	− 0.062 (− 0.240; 0.116)
	Hippocampus	− 0.046 (− 0.112; 0.020)	− 0.134 (− 0.270; 0.003)
	Amygdala	− 0.057 (− 0.152; 0.038)	− 0.136 (− 0.334; 0.063)
	Thalamus	0.043 (− 0.023; 0.109)	0.029 (− 0.107; 0.165)
Z-drugs	Total brain	0.008 (− 0.035; 0.052)	− 0.001 (− 0.099; 0.096)
	Grey matter	0.044 (− 0.068; 0.156)	0.092 (− 0.170; 0.355)
	White matter	− 0.035 (− 0.133; 0.064)	− 0.091 (− 0.317; 0.135)
	Hippocampus	− 0.019 (− 0.098; 0.061)	0.068 (− 0.112; 0.247)
	Amygdala	0.052 (− 0.058; 0.163)	0.072 (− 0.183; 0.327)
	Thalamus	− 0.004 (− 0.081; 0.073)	− 0.068 (− 0.242; 0.105)

Effect estimates reflect the difference in change in standardized brain volume per 1-year follow-up compared to never users. Model is adjusted for age, sex, education, time between inception of pharmacy records and scan date, smoking status, alcohol use, estimated glomerular filtration rate, fat mass, and prevalence of depression, diabetes, hypertension, sleep problems, coronary heart disease, heart failure, atrial fibrillation, cancer, chronic obstructive pulmonary disease, and stroke. *CI*, confidence interval; *DDD*, defined daily dose

## Discussion

In this prospective population-based study, benzodiazepine use was not associated with increased dementia risk. However, we did observe associations with accelerated reduction in hippocampal and to a lesser extent amygdalar volume over time. Yet, we found no evidence of a clear and consistent dose–response relationship, and subgroup analyses did not support stronger associations for benzodiazepines with potentially more harmful pharmacodynamic properties.

The absence of an association between benzodiazepine use and dementia risk contradicts findings from recent meta-analyses of previous research, which showed an association between benzodiazepine use and increased risk of dementia with pooled odds ratios of 1.33 to 1.78 [8, 9]. The discrepancy might be attributed to variation in study design. Sensitivity analyses within the meta-analyses suggested that observed associations may have been influenced by reversed causation and confounding by indication, with no significant associations and smaller effect estimates in studies that more adequately accounted for these types of bias [9]. Moreover, publication bias may have skewed meta-analysis towards positive associations. In the current study, we attempted to minimize reversed causation by excluding participants with cognitive impairment at baseline and assessing benzodiazepine use until baseline rather than until dementia diagnosis. When including benzodiazepine exposure in a time-varying manner up to dementia diagnosis as a sensitivity analysis, we indeed observed a positive association.

A high cumulative dose of anxiolytics was associated with an increased risk of dementia; similar results were observed in Z-drug users, while no such association was observed among sedative-hypnotics users. The primary distinction between anxiolytics and sedative-hypnotics is based on pharmacokinetics. Sedative-hypnotics, prescribed against sleep problems, primarily consist of benzodiazepines with short half-life to minimize daytime drowsiness, while anxiolytics, prescribed against anxiety, contain benzodiazepines with long half-life to provide sustained effects throughout the day. We did not observe differences in effect estimates between long half-life and short half-life anxiolytics, indicating that the half-life of benzodiazepine does not impact the observed effect. Stratification on indication revealed higher effect estimates among users with existing anxiety disorders, suggesting that confounding by indication might have influenced these results, although we observed elevated effect estimates with high cumulative doses of Z-drugs, typically prescribed against sleep problems.

Given the availability of effective alternative pharmacological and non-pharmacological treatments for anxiety and sleep problems [25], it is important to carefully consider the necessity of prolonged benzodiazepine use in light of potential detrimental effects on brain health. In our study, we observed an association between benzodiazepine use and subclinical accelerated reduction in hippocampal volume, which is in contrast with one earlier performed cross-sectional study, which reported *larger* hippocampal volumes in benzodiazepine users [14]. While this cross-sectional study was performed within a memory-clinic population, our study is the first to focus on long-term associations within a cognitively healthy group. Although the exact mechanisms underlying the effect of benzodiazepines on brain volume are still unclear, one animal study showed decreased neuronal plasticity in mice when chronically exposed to benzodiazepines [26]. In addition, benzodiazepines have suggested amyloid lowering properties [13, 27]. Several amyloid-lowering therapies have been associated with decrease in hippocampal volume without changes in clinical outcomes, of which mechanisms are still unexplained [28]. Future studies should explore whether benzodiazepine use has distinct effects on specific dementia pathologies linked to amyloid deposition in limbic structures, such as limbic-predominant age-related TDP-43 encephalopathy (LATE). Nonetheless, our result indicate that benzodiazepine use may have subtle, long-term impact on brain health, although we found no evidence of a dose–response relationship, and in contrast to dementia analyses, subgroup analyses did not support stronger effects in anxiolytics use compared to use of sedative-hypnotics. Meanwhile, our results support the current guidelines cautioning against long-term benzodiazepine prescription.

The current study is strengthened by its detailed, long-term information on benzodiazepine use as well as incident dementia and longitudinal brain imaging. There are also limitations to consider. First, the exclusion of individuals with cognitive impairment at baseline reduced reversed causation, but it may have led to selection bias and consequently underestimation of adverse effects of benzodiazepines. Second, while attrition during dementia follow-up was only 6%, one third of participants did not undergo repeated brain MRI. As participants with mental health problems or cognitive impairment are less likely to undergo repeated imaging, this might have attenuated the association between benzodiazepine use and brain atrophy. Third, all brain MRIs were conducted on the same 1.5 Tesla MRI scanner to limit inter-scanner variability and ensure consistent imaging across successive assessments, yet providing lower sensitivity to subtle structural brain changes than 3 T imaging would have. Fourth, several covariates were measured through self-report (e.g., smoking behavior, alcohol consumption, symptoms of anxiety, sleep disorder, or depression), which might be subject to information bias. Fifth, the use of DSM-III-R criteria may have led to some underdiagnosis of dementia without memory impairment [29]. Last, this study was performed in a predominantly White population, potentially hampering generalizability in view of previously reported effects of genetic and cultural differences (e.g., diet) on psychotropic medication [30].

## Conclusions

In the current study, benzodiazepine use was not associated with increased dementia risk, but potential class-dependent adverse effects and associations with subclinical markers of neurodegeneration may warrant further investigation.

### Supplementary Information

Additional file 1: Figure S1. Overview of the study design and inclusion criteria.

Additional file 2: Table S1. Dementia risk stratified by presence of anxiety, poor sleep, depressive symptoms and alcohol use.

Additional file 3: Table S2. Benzodiazepine use and standardized brain volumes at baseline.

Additional file 4: Table S3. Benzodiazepine use (any type) and change in standardized brain volumes per year during follow-up.

## Data Availability

The datasets used and/or analyzed during the current study are available from the corresponding author on reasonable request. Requests can be directed to the secretariat of the Department of Epidemiology (secretariat.epi@erasmusmc.nl), or visit the following website for more information http://www.ergo-onderzoek.nl/wp/contact.

## Abbreviations

ATC code: Anatomical Therapeutic Chemical code
BDNF: Brain-derived neurotropic factor
CAMDEX: Cambridge Examination for Mental Disorders of the Elderly
CES-D: Center for Epidemiology Depression Scale
CI: Confidence interval
CIDI: Composite International Diagnostic Interview
DDD: Defined Daily Dosage
DSM: Diagnostic and Statistical Manual of Mental Disorders
DXA: Dual-energy X-ray absorptiometry
eGFR: Estimated glomerular filtration rate
FLAIR: Fluid-attenuated inversion recovery
GMS: Geriatric Mental Schedule
HR: Hazard ratio
LATE: Limbic-predominant Age-related TDP-43 Encephalopathy
MMSE: Mini-Mental State Examination
MRI: Magnetic resonance imaging
PSQI: Pittsburg Sleep Quality Index
RS: Rotterdam Study
