# Supplementary material for: Benzodiazepine use in relation to long-term dementia risk and imaging markers of neurodegeneration: a population-based study
Source: BMC Med. 2024 Jul 2;22:266. doi: 10.1186/s12916-024-03437-5 (PMC11218055; doi:10.1186/s12916-024-03437-5)
Supplement: Supplementary file 1 — Additional file 1: Figure S1. Overview of the study design and inclusion criteria. [file 12916_2024_3437_MOESM1_ESM.pdf]

Figure S1. Overview of the study design and inclusion criteria.

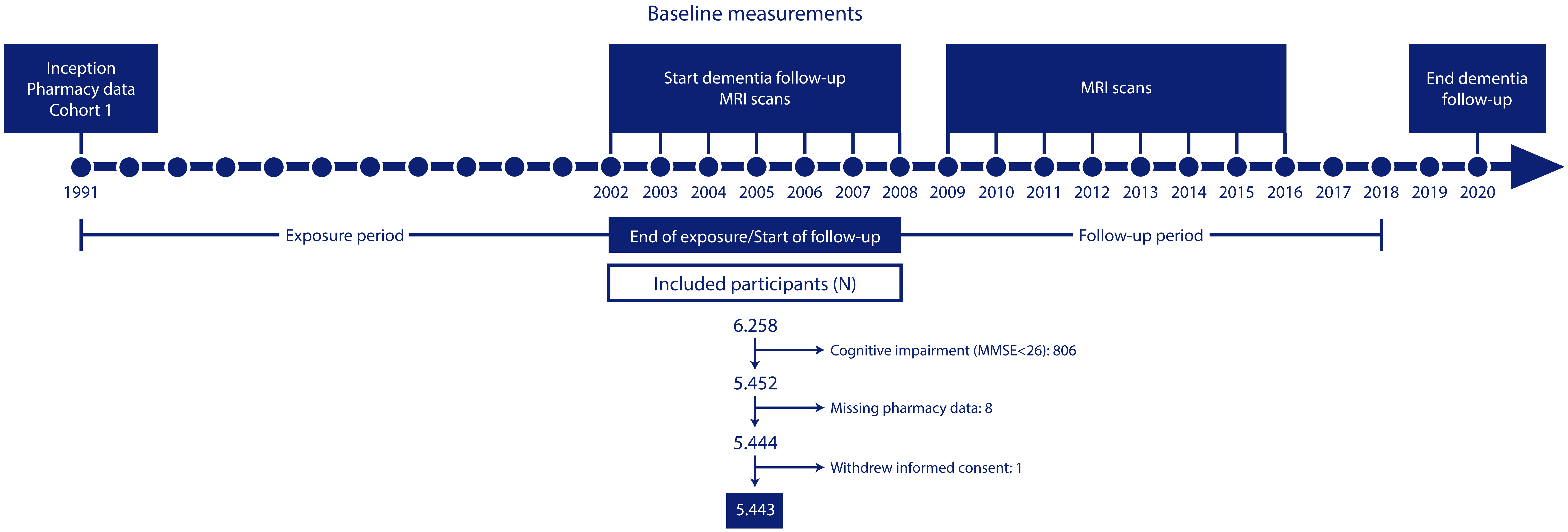

Follow-up for dementia was complete for 93.9% of the potential person years.
