# Supplementary material for: Benzodiazepine use in relation to long-term dementia risk and imaging markers of neurodegeneration: a population-based study
Source: BMC Med. 2024 Jul 2;22:266. doi: 10.1186/s12916-024-03437-5 (PMC11218055; doi:10.1186/s12916-024-03437-5)
Supplement: Supplementary file 2 — Additional file 2: Table S1. Dementia risk stratified by presence of anxiety, poor sleep, depressive symptoms and alcohol use. [file 12916_2024_3437_MOESM2_ESM.docx]

| **Table S1. Dementia risk stratified by presence of anxiety, poor sleep, depressive symptoms and alcohol use** | | | | | | | | | | | |
| --- | --- | --- | --- | --- | --- | --- | --- | --- | --- | --- | --- |
|  | **Anxiety** | | |  | **Poor sleep** | | |  | **Depressive symptoms** | | |
|  | Prevalence | Not present | Present |  | Prevalence | Not present | Present |  | Prevalence | Not present | Present |
|  |  | HR (95% CI) | HR (95% CI) |  |  | HR (95% CI) | HR (95% CI) |  |  | HR (95% CI) | HR (95% CI) |
| Never use | 124 (4.7%) | *Reference* | *Reference* |  | 491 (18.5%) | *Reference* | *Reference* |  | 114 (4.3%) | *Reference* | *Reference* |
| Ever use | 267 (10.4%) | 1.05 (0.88-1.25) | 1.47 (0.78-2.79) |  | 1038 (39.9%) | 1.19 (0.98- 1.45) | 0.86 (0.63-1.16) |  | 331 (13.0%) | 1.06 (0.89-1.26 | 1.03 (0.59-1.81) |
| Former use | 219 (9.8%) | 1.07 (0.90;1.28) | 1.24 (0.63-2.41) |  | 814 (35.9%) | 1.20 (0.99-1.47) | 0.86 (0.63-1.17) |  | 254 (11.4%) | 1.09 (0.91-1.30) | 0.98 (0.55-1.75) |
| Current use | 48 (15.0%) | 0.87 (0.60;1.26) | 2.99 (1.19-7.52) |  | 224 (66.7%) | 1.14 (0.68-1.91) | 0.82 (0.53-1.26) |  | 77 (23.9%) | 0.91 (0.63-1.31) | 1.25 (0.59-2.61) |
| Model is adjusted for age, sex, education, time between inception of pharmacy records and baseline visit, smoking status, alcohol use, estimated glomerular filtration rate, fat mass, and prevalence of depression, diabetes, hypertension, sleep problems, coronary heart disease, heart failure, atrial fibrillation, cancer, chronic obstructive pulmonary disease and stroke. HR = hazard ratio. CI = confidence interval. No use of any type of benzodiazepines is used as reference throughout. | | | | | | | | | | | |

| **Table S1. Dementia risk stratified by presence of anxiety, poor sleep, depressive symptoms and alcohol use (continuation)** | | | |
| --- | --- | --- | --- |
|  |  | **Alcohol use** |  |
|  | Prevalence | Low use  (< 2 units) | High use  (>= 2 units) |
|  |  | HR (95%CI) | HR(95%CI) |
| Never use | 710 (26.4%) | Reference | Reference |
| Ever use | 630 (23.7%) | 1.06 (0.88 – 1.28) | 1.08 (0.73 – 1.59) |
| Former use | 567 (24.6%) | 1.07 (0.89 – 1.29) | 1.09 (0.74 – 1.61) |
| Current use | 63 (18.4%) | 1.02 (0.72 – 1.44) | 0.85 (0.34 – 2.15 |
| Model is adjusted for age, sex, education, time between inception of pharmacy records and baseline visit, smoking status, alcohol use, estimated glomerular filtration rate, fat mass, and prevalence of depression, diabetes, hypertension, sleep problems, coronary heart disease, heart failure, atrial fibrillation, cancer, chronic obstructive pulmonary disease and stroke. HR = hazard ratio. CI = confidence interval. No use of any type of benzodiazepines is used as reference throughout. | | | |
