# Supplementary material for: Benzodiazepine use in relation to long-term dementia risk and imaging markers of neurodegeneration: a population-based study
Source: BMC Med. 2024 Jul 2;22:266. doi: 10.1186/s12916-024-03437-5 (PMC11218055; doi:10.1186/s12916-024-03437-5)
Supplement: Supplementary file 3 — Additional file 3: Table S2. Benzodiazepine use and standardized brain volumes at baseline. [file 12916_2024_3437_MOESM3_ESM.docx]

| **Table S2. Benzodiazepine use and standardized brain volumes at baseline** | | | |
| --- | --- | --- | --- |
| **Brain region** |  |  | **Mean difference**  **(95% CI)** |
| **Total brain** | Ever use |  | 0.001 (-0.018 ; 0.021) |
|  | Cumulative DDD | < median | 0.004 (-0.018 ; 0.025) |
|  |  | >= median | -0.003 (-0.030 ; 0.024) |
|  | Past or current use | past | 0.005 (-0.014 ; 0.025) |
|  |  | current | -0.053 (-0.103 ; -0.003) |
|  |  |  |  |
| **Grey matter** | Ever use |  | 0.005 (-0.028 ; 0.037) |
|  | Cumulative DDD | < median | 0.008 (-0.030 ; 0.046) |
|  |  | >= median | -0.001 (-0.042 ; 0.040) |
|  | Past or current use | Past | 0.009 (-0.023 ; 0.042) |
|  |  | Current | -0.062 (-0.144 ; 0.020) |
|  |  |  |  |
| **White matter** | Ever use |  | -0.002 (-0.036 ; 0.032) |
|  | Cumulative DDD | < median | -0.006 (-0.046 ; 0.034) |
|  |  | >= median | 0.006 (-0.037 ; 0.049) |
|  | Past or current use | Past | 0.000 (-0.034 ; 0.035) |
|  |  | Current | -0.032 (-0.119 ; 0.054) |
|  |  |  |  |
| **Hippocampus** | Ever use |  | -0.018 (-0.060 ; 0.024) |
|  | Cumulative DDD | < median | -0.026 (-0.075 ; 0.024) |
|  |  | >= median | -0.005 (-0.058 ; 0.049) |
|  | Past or current use | Past | -0.010 (-0.052 ; 0.033) |
|  |  | Current | -0.137 (-0.244 ; -0.030) |
|  |  |  |  |
| **Amygdala** | Ever use |  | -0.012 (-0.054 ; 0.031) |
|  | Cumulative DDD | < median | -0.012 (-0.062 ; 0.038) |
|  |  | >= median | -0.009 (-0.062 ; 0.045) |
|  | Past or current use | Past | -0.002 (-0.044 ; 0.041) |
|  |  | Current | -0.163 (-0.270 ; -0.056) |
|  |  |  |  |
| **Thalamus** | Ever use |  | -0.021 (-0.059 ; 0.018) |
|  | Cumulative DDD | < median | -0.003 (-0.048 ; 0.042) |
|  |  | >= median | 0.042 (-0.091 ; 0.007) |
|  | Past or current use | Past | -0.011 (-0.050 ; 0.027) |
|  |  | Current | -0.160 (-0.258 ; -0.063) |
| Mean difference represents mean difference in standardized brain volumes at baseline. All estimates refer to the comparison with never-use. Model is adjusted for age, sex, education, time between inception of pharmacy records and scan date, smoking status, alcohol use, estimated glomerular filtration rate, fat mass, and prevalence of depression, anxiety disorders, sleep problems, diabetes, hypertension, coronary heart disease, heart failure, atrial fibrillation, cancer, chronic obstructive pulmonary disease and stroke. CI = confidence interval. DDD = defined daily dose. | | | |
