# Supplementary material for: Benzodiazepine use in relation to long-term dementia risk and imaging markers of neurodegeneration: a population-based study
Source: BMC Med. 2024 Jul 2;22:266. doi: 10.1186/s12916-024-03437-5 (PMC11218055; doi:10.1186/s12916-024-03437-5)
Supplement: Supplementary file 4 — Additional file 4: Table S3. Benzodiazepine use (any type) and change in standardized brain volumes per year during follow-up. [file 12916_2024_3437_MOESM4_ESM.docx]

| **Table S3**. **Benzodiazepine use (any type) and change in standardized brain volumes per year during follow-up** | | | | |
| --- | --- | --- | --- | --- |
| **Brain region** |  |  | **1^st^ spline:**  **0 - 3 year of follow-up** | **2^nd^ spline:**  **3 - 10 year of follow-up** |
|  |  |  | Mean difference  (95% CI) | Mean difference  (95% CI) |
| Total brain | Cumulative DDD | < median | 0.004 (-0.024 ; 0.033) | -0.020 (-0.085 ; 0.045) |
|  |  | >= median | -0.000 (-0.030 ; 0.029) | -0.002 (-0.066 ; 0.062) |
|  | Past or current use | Past | 0.002 (-0.022 ; 0.026) | -0.016 (-0.069 ; 0.036) |
|  |  | Current | 0.001 (-0.066 ; 0.068) | 0.028 (-0.114 ; 0.170) |
|  |  |  |  |  |
| Grey matter | Cumulative DDD | < median | -0.055 (-0.129 ; 0.018) | -0.117 (-0.293 ; 0.059) |
|  |  | >= median | 0.045 (-0.031 ; 0.121) | 0.030 (-0.141 ; 0.201) |
|  | Past or current use | Past | -0.021 (-0.082 ; 0.040) | -0.053 (-0.194 ; 0.087) |
|  |  | Current | 0.153 (-0.019 ; 0.324) | 0.100 (-0.278 ; 0.479) |
|  |  |  |  |  |
| White matter | Cumulative DDD | < median | 0.058 (-0.007 ; 0.123) | 0.075 (-0.076 ; 0.226) |
|  |  | >= median | -0.038 (-0.105 ; 0.030) | -0.016 (-0.164 ; 0.132) |
|  | Past or current use | Past | 0.024 (-0.030 ; 0.078) | 0.031 (-0.090 ; 0.152) |
|  |  | Current | -0.123 (-0.275 ; 0.029) | -0.028 (-0.356 ; 0.301) |
|  |  |  |  |  |
| Hippocampus | Cumulative DDD | < median | -0.009 (-0.061 ; 0.043) | -0.149 (-0.269 ; -0.028) |
|  |  | >= median | -0.028 (-0.082 ; 0.026) | -0.090 (-0.207 ; 0.028) |
|  | Past or current use | Past | -0.021 (-0.064 ; 0.022) | -0.125 (-0.221 ; -0.028) |
|  |  | Current | 0.017 (-0.105 ; 0.138) | -0.027 (-0.288 ; 0.233) |
|  |  |  |  |  |
| Amygdala | Cumulative DDD | < median | -0.027 (-0.100 ; 0.045) | -0.146 (-0.317 ; 0.025) |
|  |  | >= median | -0.047 (-0.122 ; 0.028) | -0.074 (-0.241 ; 0.093) |
|  | Past or current use | Past | -0.043 (-0.103 ; 0.017) | -0.131 (-0.268 ; 0.005) |
|  |  | Current | 0.065 (-0.105 ; 0.234) | 0.226 (-0.143 ; 0.595) |
|  |  |  |  |  |
| Thalamus | Cumulative DDD | < median | -0.002 (-0.052 ; 0.049) | 0.027 (-0.090 ; 0.143) |
|  |  | >= median | 0.048 (-0.004 ; 0.101) | 0.032 (-0.082 ; 0.146) |
|  | Past or current use | Past | 0.020 (-0.022 ; 0.062) | 0.017 (-0.077 ; 0.110) |
|  |  | Current | 0.017 (-0.102 ; 0.135) | 0.069 (-0.185 ; 0.322) |
| Effect estimates reflect the change in standardized brain volume per 1-year follow-up compared to never users. Model is adjusted for age, sex, education, smoking status, alcohol use, estimated glomerular filtration rate, fat mass, and prevalence of depression, diabetes, hypertension, sleep problems, coronary heart disease, heart failure, atrial fibrillation, cancer, chronic obstructive pulmonary disease and stroke. CI = confidence interval. DDD = defined daily dose. No use of any type of benzodiazepines is used as reference throughout. | | | | |
